# Equilibrio ácido-base y COVID largo: comentarios sobre las alteraciones metabólico-respiratorias

**DOI:** 10.31053/1853.0605.v80.n4.42580

**Published:** 2023-12-26

**Authors:** Brian Johan Bustos-Viviescas, Rafael Enrique Lozano Zapata, Carlos Enrique García Yerena

**Affiliations:** 1 Centro de Comercio y Servicios, SENA Regional Risaralda. Pereira, Colombia Pereira Colombia; 2 Universidad de Pamplona Villa del Rosario Colombia; 3 Universidad del Magdalena Santa Marta Colombia

**Estimado director:**

Alrededor del mundo se calcula que aproximadamente 65 millones tienen COVID largo, estas cifras seguirán aumentando progresivamente con el tiempo debido a la facilidad de propagación del virus, ^1^ de acuerdo a estas circunstancias el COVID se ha convertido en una problemática para la salud pública a nivel mundial, donde diferentes organismos han recaudado información valiosa sobre esta enfermedad, determinando sus causas, llevando a cabo un tratamiento y rehabilitación eficiente para reducir estas cifras; lo anterior mencionado, es con el objetivo que la población objeto de estudio pueda llevar una vida más tranquila y sana. ^2^

Cabe resaltar que, de acuerdo a esta enfermedad por infección de COVID 19, quienes la han padecido se pueda reflejar situaciones que implican fatiga crónica al realizar actividad física, pese a que no se presenten arritmias cardiacas o afecciones pulmonares, afectando la función mitocondrial y con esto, generando información valiosa a nivel clínico para estos pacientes con COVID largo, ^3^ por lo que, la comprensión de los cambios en la función mitocondrial como es el caso del equilibrio ácido-base metabólico y respiratorio, permitirá estructurar, aplicar y valorar estrategias de gestión y tratamiento más eficaces para los pacientes con COVID largo.

A través de la literatura científica, se encuentra un estudio con sobrevivientes y no sobrevivientes de enfermedades críticas tempranas, donde se evidenciaron aumentos a nivel celular de estrés oxidativo, y a su vez, impulso reductivo. En un principio, los pacientes que no lograron superar la enfermedad y fallecieron se les encontró mayor capacidad antioxidante, al igual, mayor participación lipoperoxidación con relación a los pacientes que sí lograron sobrevivir. ^4^

Ahora bien, en pacientes ingresados con COVID-19 las investigaciones previas han notificado variaciones destacables del pH, encontrando alteraciones a nivel metabólico como respiratoria, con una marcada alcalosis, ^5^ del mismo modo, una brecha aniónica alta, generando una acidosis metabólica, ^6^ por otro lado, al observar estas alteraciones a nivel metabólico y respiratorio, se reflejaron mayor riesgo de mortalidad en estos pacientes. ^7^

Diferentes trabajos han identificado niveles altos de metabolitos o enzimas de la glucólisis, esto sugiere que el metabolismo glucolítico es un factor crucial para el avance del SARS-CoV-2, (8-11) teniendo en cuenta esto, la influencia de NAD al ser regulados durante su funcionamiento metabólico, puede generar beneficios terapéuticos, esto, al momento de desarrollar un cambio en la enfermedad de COVID-19, ^12^ de esta manera, dicha enfermedad permite que se tengan alteraciones metabólicas, de forma permanente y repetitivas, a su vez estableciendo disfunción mitocondrial, facilitando de forma continua inflamaciones y cambios en la glucólisis. ^13^

Como mecanismos de asistencia para encontrar beneficios con pacientes post COVID, la oxigenoterapia hiperbárica es uno de ellos, la cual mostró una mejor funcionalidad a nivel de fuerza, resistencia y mayor calidad de vida, a su vez mejora los parámetros respiratorios y de acidosis metabólica, ^14^ es por ello que un tratamiento adecuado, puede generar beneficios a nivel metabólico, y reducir las diferentes situaciones que se presentan durante la enfermedad, esto al ser suministrado por vía intravenosa NAD+, como medio terapéutico y dar un manejo apropiado a los síntomas del síndrome postagudo del COVID-19. ^15^

Es importante destacar que, los pacientes con infección por SARS-CoV-2, que han estado hospitalizados con neumonía, generan dificultades en cualquier parte del organismo, dentro de las cuales se encuentra, la insuficiencia respiratoria (71,14%), hiperglucemia (43,54%), lesión renal (42,67%) o eventos cardiovasculares (7,10%), de esta manera, se ha convertido en un problema clínico, debido a las dificultades que se generan al tratar de forma eficiente estos pacientes, ^16^ por ello, los sobrevivientes que presentaban complicaciones respiratorias previas al COVID-19 como neumonía, son actualmente una de las poblaciones de riesgo elevado a los síntomas del COVID largo, y, se requiere la evaluación en futuras investigaciones del COVID largo con respecto al equilibrio ácido-base.

El COVID largo es una patología multiorgánica con secuelas importantes que afectan la salud y bienestar de los sobrevivientes desde la bioenergética celular, en este caso, se abordó la importancia de considerar el equilibro ácido-base dentro de los procesos de evaluación y tratamiento del COVID largo por parte de los profesionales de la salud y el deporte, dado a que, diferentes investigaciones han encontrado modificaciones importantes en la función mitocondrial que se traducen en fallas de la ventilación, esto provocando alteraciones de la compensación-descompensación del control respiratorio y renal del pH (acidosis y alcalosis metabólico-respiratorias).
